# Ocular adnexal lymphoma – a retrospective study and review of the literature

**DOI:** 10.2478/raon-2024-0048

**Published:** 2024-09-15

**Authors:** Lucka Boltezar, Danijela Strbac, Joze Pizem, Gregor Hawlina

**Affiliations:** Department of Medical Oncology, Institute of Oncology Ljubljana, Ljubljana, Slovenia; Faculty of Medicine, University of Ljubljana, Ljubljana, Slovenia; Department of Radiotherapy, Institute of Oncology Ljubljana, Ljubljana, Slovenia; Institute of Pathology, Faculty of Medicine, University of Ljubljana, Ljubljana, Slovenia; Eye Hospital, University Medical Centre Ljubljana, Ljubljana, Slovenia

**Keywords:** ocular adnexal lymphoma, orbital lymphoma, conjunctival lymphoma, MALT lymphoma, lymphoma treatment

## Abstract

**Background:**

To review the characteristics of all Slovenian patients with ocular adnexal lymphoma (OAL) in the period of 24 years with the aim of evaluating demographic data, lymphoma location and type, disease stage, treatment modality, local control rate and survival rate.

**Patients and methods:**

All patients with histologically diagnosed OAL in the main tertiary centre of Slovenia, Eye Hospital, University Medical Centre Ljubljana, who were treated at Institute of Oncology Ljubljana were included in the study. Patients’ data were collected from October 1995 through April 2019.

**Results:**

Seventy-four patients were included in the study having a median age of 68 years at diagnosis. The majority of lymphomas were of B-cell origin (98.6%). The most frequent type was the extranodal marginal zone B-cell lymphoma (MALT) (71.6%). Orbital lymphomas were diagnosed in 56 cases (75.7%) and conjunctival in 18 cases (24.3%). Ocular manifestation was the first sign of the disease in 78.4% of patients and in 67.6% of patients ocular adnexa were the only disease location. Fifty-one patients (68.9%) were treated with radiotherapy, 7 patients (9.4%) with systemic treatment, 5 patients (6.8%) with combined radiotherapy and systemic treatment and in 11 patients, biopsy and active surveillance strategy was applied (14.9%). Local control of the disease was achieved in 96.6% of treated patients. Median overall survival of the whole study group has not been reached yet. Five-year overall survival rate was 80.1% (95% CI 68.1% – 88.5%) and 5-year lymphoma specific survival rate was 87.2% (95% CI 83.2%−91.2%).

**Conclusions:**

OALs comprise a group of heterogeneous diseases with variable outcomes depending predominately on the patient’s age and lymphoma type, with low grade lymphomas carrying good prognosis even in elderly patients.

## Introduction

Lymphomas are a heterogeneous group of malignant lymphoid tumors that arise from the clonal proliferation of either B-lymphocytes, T-lymphocytes or, less commonly, of natural killer (NK) cells at different stages of maturation.^1,2^ Lymphomas are divided into 2 major categories, namely Hodgkin lymphoma (HL) and non-Hodgkin lymphoma (NHL). The molecular mechanisms include several structural chromosomal abnormalities, for example translocation 14;18 in marginal zone lymphomas, translocation 11;14 in mantle cell lymphomas or bcl-2, bcl-6 and myc translocation in case of aggressive B-cell lymphomas.^1,2,3,4,5^ Ocular adnexal lymphoma (OAL) is defined as lymphoma that occurs in the conjunctiva, lacrimal apparatus, eyelid, or orbit.^3,4,5,6^ It represents 6−8% of orbital and 10−15% of adnexal tumors.^7^ OAL is considered primarily if it involves the ocular adnexa alone and secondarily if it is accompanied by a lymphoma of the identical type at another extraocular site.^6^ These tumors may spread locally or disseminate systemically.^8^ OAL may present with symptoms of conjunctival salmon patches, ptosis from levator muscle involvement or the insidious and painless development of proptosis with or without diplopia due to an orbital mass.^9^ In the orbit, OAL can involve the lacrimal gland, extraocular muscles, or intraconal and extraconal spaces.^9^

Diagnosis based only on clinical and imaging data is inadequate. Consequently, an incisional or excisional biopsy followed by histopathologic, immunophenotypic and molecular genetic studies, either to confirm or to rule out this malignancy, should be performed.^10^ Orbital imaging typically shows a poorly defined mass that molds to the shape of surrounding structures without direct invasion or bony erosion.^11,12^ In addition to orbital imaging and subsequent biopsy, positron emission tomography alone or combined with computed tomography is often performed for systemic staging of disease and for the assessment of response to therapy.^13^ It is important to perform a complete systemic evaluation, both at diagnosis and at regular follow-ups, since more than half of patients will present with systemic lymphoma at the time of ocular diagnosis or will develop a systemic disease later on.^14^

Several successful treatment modalities for OAL have been reported so far, including surgical excision, cryotherapy, external beam radiotherapy for tumors localized to the periocular area or in combination with chemotherapy, immune modulating therapy, or primary antibiotic treatment in case of systemic involvement.^15^ Spontaneous remissions of the OAL have been reported in low grade lymphomas but never in high grade subtypes. In general, the outcome of OAL is favourable, however small patient numbers, patient selection criteria, varied histologic subtypes, and the lack of large prospective studies usually make the comparison of the effectiveness of different types of treatment challenging.^16^

The purpose of our study was to review the characteristics of all Slovenian patients with OAL in the period of 24 years with the aim of evaluating demographic data, lymphoma location and type, disease stage, treatment modality, local control rate and survival rate.

## Patients and methods

### Patients

This retrospective study included all patients with histologically diagnosed OAL in the main tertiary centre of Slovenia Eye Hospital, University Medical Centre Ljubljana, who continued their treatment at the Institute of Oncology Ljubljana. Patients’ data were collected from October 1995 through April 2019. The primary end points were lymphoma type, lymphoma location, demographic data and survival rate. Data regarding patient’s age, stage of disease, treatment modality, treatment outcome and survival rate were gathered from their electronic records. Survival data were obtained from the Cancer Registry of Republic of Slovenia. Staging was performed according to the guidelines^17^ using the Ann Arbor staging system.^18^ All patients were treated at Institute of Oncology Ljubljana, the treatment decision for each individual patient was consented at the lymphoma tumor board and patients were treated in accordance with the National lymphoma guidelines^19^, which are updated on yearly basis. For low grade lymphomas the active surveillance strategy or radiotherapy treatment was proposed, depending on the comorbidities of each patient and symptoms of the OAL. For high grade lymphomas the treatment strategy was systemic treatment, sometimes combined with radiotherapy in case of high burden of ocular symptoms.

This study was approved by Institutional Review Board and Institutional Ethical Committee (ERIDNPVO-0008/2022, 2.12.2021 and 16.12.2021) and was executed according to the declaration of Helsinki. All patients signed an informed consent form to participate in the study.

### Clinical characteristics

#### Symptoms and signs

Our patients presented with symptoms such as decreased visual acuity, diplopia, tearing or retrobulbar pain and with signs as lid mass, proptosis, ptosis, lacrimal gland mass, chemosis or conjunctival injection, limitation of extraocular movement, enlarged lacrimal sac and globe displacement. After initial full ophthalmological examination, blood tests and orbital imaging including ultrasound (US), computed tomography (CT) and/or magnetic resonance imaging (MRI) were performed. Subsequently, incisional biopsy was performed in most of the cases while in cases where lesion was small and localized, an excisional biopsy was carried out.

#### Lymphoma location

**FIGURE 1.**
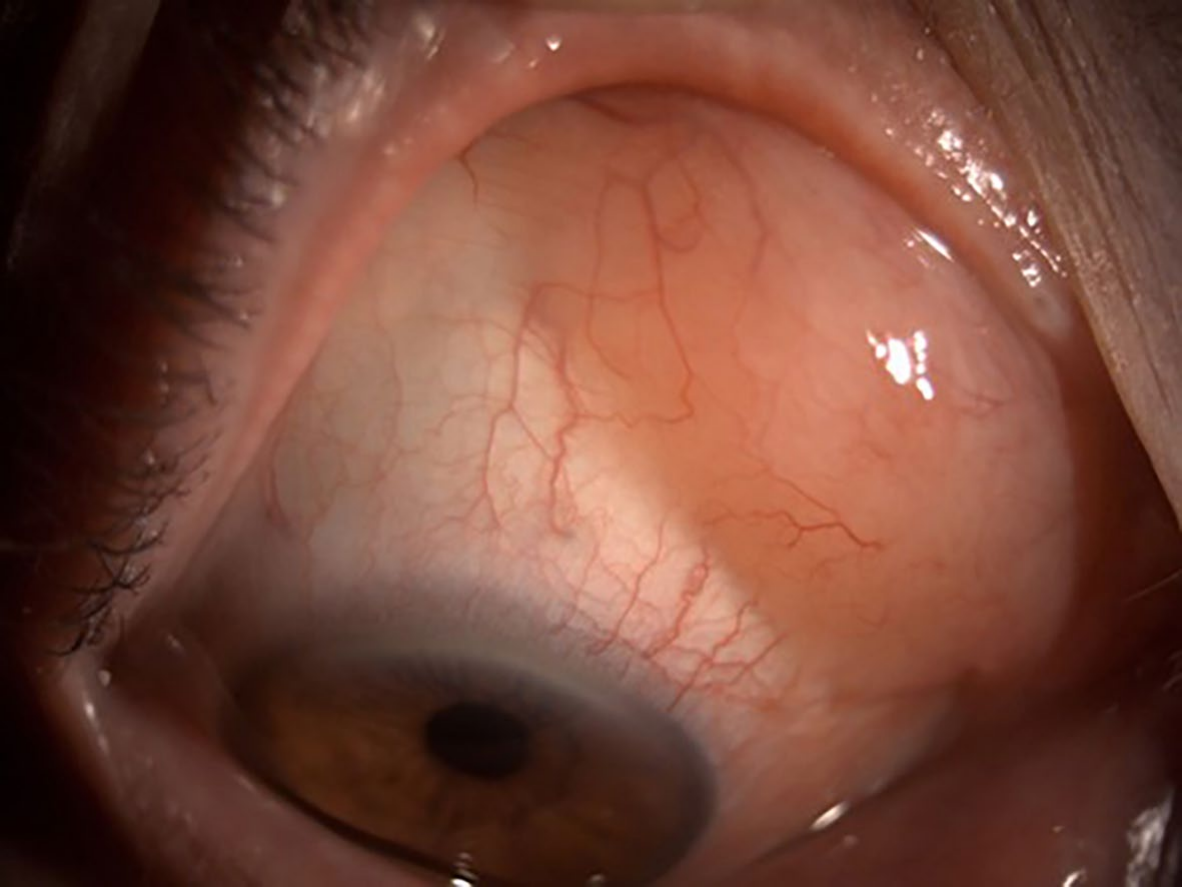
Patient with conjunctival lymphoma presents with a characteristic salmon-pink nodular patch in the conjunctiva.

**FIGURE 2.**
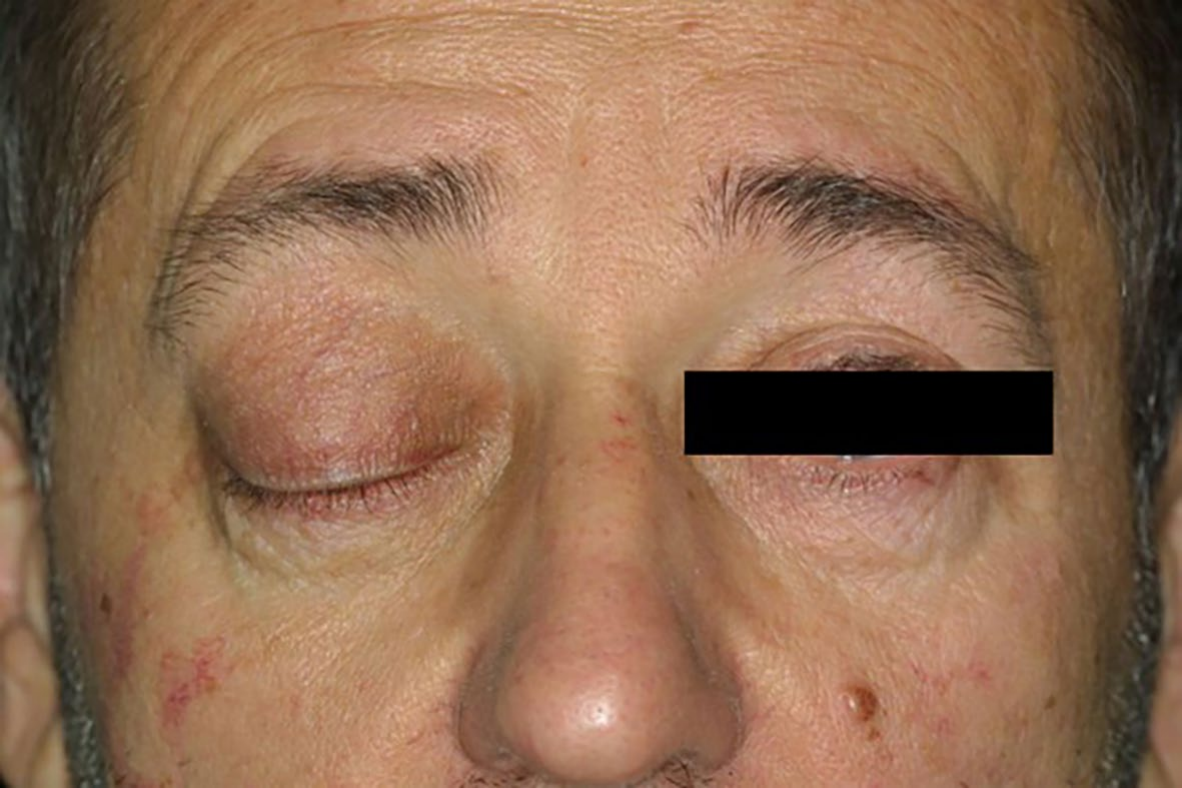
Patient with orbital lymphoma presents with a mass lesion within the right eye.

Patients with OAL were divided according to the location of lymphoma into orbital and conjunctival lymphoma groups. Conjunctival lymphoma presented as a characteristic salmon-pink nodular patch in the conjunctiva (Figure 1), while orbital lymphoma presented with a mass lesion in eyelids, lacrimal gland, lacrimal sac, extraocular muscle, intraconal or extraconal space (Figure 2).

Locations of the OAL were navigated by patients’ physical examination and radiology imaging (CT, MRI and/or US). In conjunctival lymphomas, the biopsy was performed transconjunctivally and specimen included part of the conjunctiva while in orbital lymphoma the biopsy was performed with different anterior orbitotomy approaches. Lymphoma was staged according to the guidelines^17^ with a CT of the neck, thorax and abdomen in marginal zone B-cell lymphoma and with positron emission tomography-CT (PET-CT) when other, more aggressive histological subtypes were present.

#### Histopathologic examination

All biopsy samples were fixed in formalin and embedded in paraffin. In addition to morphological criteria, appropriate immunohistochemical markers were applied for the diagnosis of different types of lymphomas.^20^ The following lymphoma types mostly of B-cell origin have been identifed: extranodal marginal zone lymphoma (MALT), follicular lymphoma (FL), mantle cell lymphoma (MCL), chronic lymphocytic leukemia (CLL), diffuse large B-cell lymphoma (DLBCL), together with the peripheral T-cell lymphoma, not otherwise specified.

### Statistical analyses

For demographic data, descriptive statistics was used. For numeric variables a non parametric t-test was used (Mann-Whitney). Survival was estimated using the Kaplan-Meier method. Overall survival was defined as time from histological diagnosis until death from any reason or censoring on 14.11.2022. Lymphoma specific survival was defined as time from histological diagnosis until death, caused by or related to lymphoma.

## Results

### Demographics

A total of 74 patients with a histologically verified lymphoma of the orbit (i.e. eyelid, lacrimal gland, lacrimal sac, and/or rectus muscles) or conjunctiva were included. There were 40.5% male and 59.5% female patients. Median age at OAL diagnosis for the whole group was 68 years (range 34 – 89 years). In 58 patients (78.4%), the histological verification was done at the first disease presentation and in 16 patients (21.6%) it was done at recurrent presentation in ocular adnexa. Out of 58 patients with a biopsy at the first disease presentation, 13 patients (22.4%) were subsequently diagnosed with a systemic lymphoma according to radiological staging. Patients’ characteristics are presented in Table 1.

**TABLE 1.** Patients’ characteristics

	**Males**	**Females**	**All**
**Number of patients**	30 (40.5%)	44 (59.5%)	74
**Median age, years**	72 (range 41 – 89)	67.5 (range 34 – 89)	68
**Lymphoma type**			
- Marginal zone lymphoma (MALT)	21/30 (70.0%)	32/44 (72.7%)	53/74 (71.6%)
- Mantle cell lymphoma (MCL)	6/30 (20.0%)	3/44 (6.8%)	9/74 (12.2%)
- Follicular lymphoma (FL)	2/30 (6.7%)	4/44 (9.0%)	6/74 (8.1%)
- Diffuse large B-cell lymphoma (DLBCL)	1/30 (3.3%)	3/44 (6.8%)	4/74 (5.4%)
- Chronic lymphocytic leukemia (CLL)	0/30 (0%)	1/44 (2.3%)	1/74 (1.4%)
- Peripheral T-cell lymphoma, not otherwise specified	0/30 (0%)	1/44 (1.4%)	1/74 (1.4%)
**Conjunctival lymphoma**	7/30 (23.3%)	11/44 (25.0%)	18/74 (24.3%)
**Orbital lymphoma**	23/30 (76.7%)	33/44 (75.0%)	56/74 (75.7%)

Median age did not differ between males and females (p=0.367). There was also no difference in the distribution of conjunctival or orbital lymphomas among males and females (p=0.870). However, all lymphoma types exhibited a female predominance except for MCL, which was more common in males.

### Anatomical location

Orbital lymphomas were diagnosed in 56 cases (75.7%) and conjunctival in 18 cases (24.3%). Orbital lymphomas appeared more frequently bilaterally than this was observed with conjunctival lymphoma (p<0.001). All lymphoma subtypes were found in the orbit, with the most frequent types being the MALT and MCL. Specifications of conjunctival and orbital lymphomas are given in Table 2.

**TABLE 2.** Specifications of orbital and conjunctival lymphomas

	**Orbital lymphomas**	**Conjunctival lymphomas**	**All**
**Unilateral lymphoma**	46/56 (82.1%)	17/18 (94.4%)	63/74 (85.1%)
**Bilateral lymphoma**	10/56 (17.9%)	1/18 (5.6%)	11/74 (14.9%)
**Lymphoma type**			
- Marginal zone lymphoma (MALT)	36/56 (64.3%)	17/18 (94.4%)	53/74 (71.6%)
- Mantle cell lymphoma (MCL)	9/56 (16.1%)	0/18 (0%)	9/74 (12.2%)
- Follicular lymphoma (FL)	5/56 (8.9%)	1/18 (5.6%)	6/74 (8.1%)
- Diffuse large B-cell lymphoma (DLBCL)	4/56 (7.1%)	0/18 (0%)	4/74 (5.4%)
- Chronic lymphocytic leukemia (CLL)	1/56 (1.8%)	0/18 (0%)	1/74 (1.4%)
- Peripheral T-cell lymphoma, not otherwise specified	1/56 (1.8%)	0/18 (0%)	1/74 (1.4%)

### Stage

Fifty patients had ocular manifestation as their only lymphoma location at the time of the biopsy (67.6%) while 24 patients (14 females and 10 males) had a systemic lymphoma (32.4%) from the beginning. Of the later, the majority had stage IV disease (87.5%, 21/24 patients), one patient had stage III disease (4.2%, 1/24) and two patients had stage II disease (8.3%, 2/24).

### Treatment

Fifty-one patients (68.9%) were treated with radiotherapy, 7 patients (9.4%) with systemic treatment, 5 patients (6.8%) with combined treatment – radiotherapy and systemic treatment and in 11 patients, biopsy and active surveillance strategy was applied (14.9%), usually on account of their advanced age and lack of symptoms. Active surveillance strategy meant that patients were regularly followed by ophtalmologist in four to six monthly periods. Patients diagnosed after 2011 and treated with radiotherapy received 24.05 Gy in 13 daily fractions and patients treated before 2011 received 30,6 Gy in 18 fractions. The techniques applied were either 3D/VMAT, 2D electrons or electron beam planning.

The systemic treatment applied was usually a combination of an anti-CD20 antibody rituximab and an anthracyclin-based regimen and all patients had an advanced stage and an aggressive subtype of lymphoma, except for one patient with an advanced MALT lymphoma. One patient underwent consolidation with autologous stem cell transplant in the first line treatment.

Out of 63 patients, who received upfront treatment with radiotherapy, systemic treatment or a combination of both, in 5 patients the evaluation of treatment was either not possible or was not performed. Treatment outcome for 58 patients with post-treatment evaluation is seen in Table 3.

**TABLE 3.** Treatment outcome in 58 patients with post-treatment evaluation, post-treatment evaluation being done three to six months after the end of treatment

**Treatment outcome**	**Number of patients**
Complete remission	46 (79.3%)
Partial remission	10 (17.2%)
Stable disease	1 (1.7%)
Progressive disease	1 (1.7%)^*^

* = Patient who progressed during first line treatment had mantle cell lymphoma (MCL)

Recurrence of lymphoma was documented in 25 of 58 patients (43.1%) during follow up (median follow up time being 72 months) while 33 of 58 treated patients remained in a long-term remission and never experienced a recurrence (56.9%). Patients were followed up regularly by the ophtalmologist and oncologist (in case of oncologic treatment) in four to six monthly periods.

Out of 25 patients experiencing a recurrence, only 2 patients had a recurrence in periocular area, that has already been treated previously (3.4%, 2/58) while 23 (92.0%, 23/25) patients had a recurrence outside the previously treated location. The two patients with a recurrence in the already treated eye had been primarily treated with radiotherapy, one with 25 Gray and the other patient with 20 Gy. The local control rate was therefore 96.6%.

For 11 patients on active surveillance strategy, six achieved complete remission of the ocular lesion spontaneously, four had stable disease and one had progression of the lesion. Recurrence of systemic lymphoma later occurred in 5 of these patients (45.5%, 5/11), however, only one patient (20.0%, 1/5) had a recurrence in the eye, which was primarily diagnosed, but not yet treated (that patient had a spontaneous remission of the disease and later on recurrence of the disease).

### Survival

Median overall survival of the whole study group has not been reached yet. Twenty-two patients were deceased at the time of the data retrieval (29.7%), however, only 11 (50.0%) of them died due to lymphoma. Median follow up time was 72 months (range 1 – 280 months). Patients, who died due to lymphoma, were: one patient with DLBCL, one patient with FL, five patients with MALT, two patients with MCL, one with CLL and one patient with peripheral T-cell lymphoma, not otherwise specified. Other patients died due to causes unrelated to lymphoma. Patients, who died due to other causes, were significantly older than the rest of the group, p=0.003, while patients, who died due to lymphoma were not older than the rest of the studied group, p=0.453.

**FIGURE 3.**
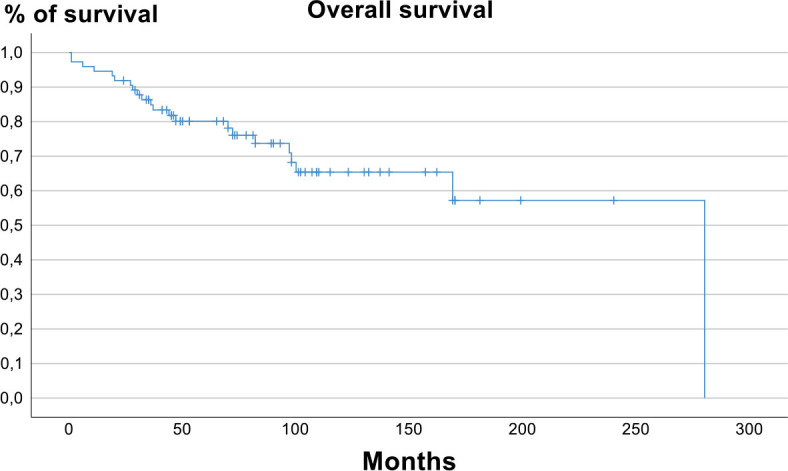
Overall survival.

**FIGURE 4.**
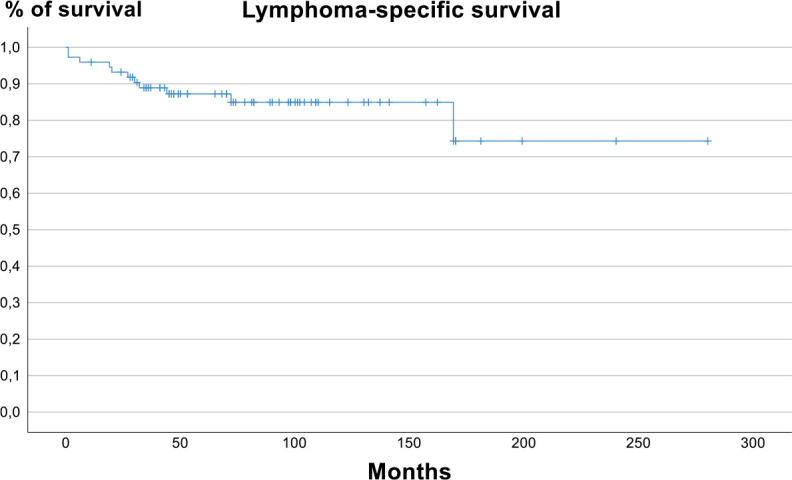
Lymphoma specific survival.

Five-year overall survival rate was 80.1% (95% CI 68.1% – 88.5%) and 5-year lymphoma specific survival rate was 87.2% (95% CI 83.2%−91.2%). Figures 3 and 4 show overall survival and lymphoma specific survival, respectively.

### Localized lymphoma subgroup

The localized lymphoma subgroup comprised 50 patients in whom no signs of systemic lymphoma could be detected at the time of the biopsy. There were 20 males (40.0%) and 30 females (60.0%). Their median age was 64 years, range 41−83 years. Conjunctival lymphomas were seen in 17 patients (34.0%) and orbital lymphomas in 33 patients (66.0%). There were 46 (92.0%) patients with MALT lymphomas, two (4.0%) patients with FL and two patients (4.0%) with MCL. All patients who were treated upfront (78.0% (39/50)) were treated with radiotherapy according to the guidelines^17^ and for 11 patients (22.0%) the active surveillance strategy was applied. In the active surveillance strategy group one patient had a FL and 10 patients had MALT lymphoma.

Five-year overall survival rate for this subgroup of patients was 91.1% (95% CI 86.9% – 95.3%) and 5-year lymphoma-specific survival was 100%. In 37 patients, the post-treatment evaluation was performed and during follow up 26 patients stayed in long term complete remission (70.3%, 26/37). Nine patients experienced a systemic relapse (24.3%, 9/37) and only two patients had a relapse in the previously treated eye (5.4%, 2/37). All patients with relapses had a MALT lymphoma.

## Discussion

In our retrospective study we present the characteristics of Slovenian patients with OAL. The study included 74 patients with biopsy-proven OAL between 1995 and 2019 at Eye Hospital, University Medical Centre Ljubljana, Slovenia, and treated at the Institute of Oncology Ljubljana, Slovenia. Compared to median age of OAL presentation of around 60 years reported in previous studies and reviews, we registered a higher median age (68 years) in our group.^16,21,22,23,24,25^ In concordance with the previous review where female predominance of 55% was reported, we also registered a higher morbidity in women (59.5%) in our study.^25^

Anatomically, our OAL cases were distributed inside the orbit in 56 cases (75.7%) compared to 18 cases (24.3%) located in conjunctiva. The distribution was similar to the study of Fernandez *et al*. where the orbital fibroadipose tissue was involved in 64% of cases and conjunctiva in 32% of cases.^22^ We anatomically divided OAL in the conjunctival lymphoma with characteristic salmon-pink nodular patch and in the orbital lymphoma with a presentation of mass lesion in eyelids, lacrimal gland, lacrimal sac, extraocular muscle, intraconal or extraconal space. In some studies, the eyelids were used as a separate location^16,22^ but we included the eyelids’ involvement with orbital lymphoma as all our patients with involved eyelids also had an orbital progression of lymphoma. Bilateral involvement of OAL is usually seen in 7%−24% of patients^16^, in our series it was in 14.9%. Bilateral involvement was, however, more common in orbital lymphoma (17.9%, 10/56) than in conjunctival lymphoma (5.6%, 1/18). Of note, bilateral OAL was detected only in MALT (4/53, 7.5%), MCL (5/9, 55.6%) and FL (2/6, 33.3%) lymphoma subtypes. According to our results, although the study included a relatively small number of MCL cases, we observed that MCL was more commonly confirmed bilaterally compared to the other types of lymphoma. The study of Rasmussen *et al*. on ocular adnexal MCL showed also a more common bilateral disease. Furthermore, they also reported a more frequent bilateral occurrence of primary than of secondary ocular adnexal MCL.^26^ On contrary, all our MCL patients had a secondary lymphoma or a recurrence of systemic lymphoma.

The majority of lymphomas in our study were of B-cell origin (98.6%, 73/74) similarly to the other studies where between 95% and 100% of reported cases corresponded to the B-cell type.^16^ Subtypes were MALT (71.6%), which is in agreement with previous studies reporting a 58% occurrence of this type^27^, MCL (12.1%), FL (8.1%), DLBCL (5.4%), CLL (1.4%) and peripheral T-cell lymphoma, not otherwise specified (1.4%). All subtypes were found in the orbit, with the most frequent being MALT (64.23%, 35/56) and MCL (16.1%, 9/56), while only MALT (94.4%, 17/18) and FL (5.6%, 1/18) were identified in conjunctiva. Comparably, conjunctival sites were observed in literature in one third of patients and almost always consisted of low-grade NHL (96% of patients).^28^ MCL was the only histological subtype which predominated in male patients, which is also consistent with previous studies^8,29,30,31^, all other histological subtypes were predominant in female patients. The only entity that stands out in our study is a relatively high proportion of MCL, 12.2%, since this type is usually diagnosed in only 3−4% of the patients.^22,24^ However, all cases of MCL in our series were located in the orbit and represented a primary or secondary manifestation of a systemic lymphoma. Further studies on larger number of patients will be needed to prove this observation.

Half of the treated patients were cured with radiotherapy or, to a minor extent, systemic treatment. Treatment guidelines suggest radiotherapy as the preferred option for localized disease in case of low-grade lymphoma.^17,19^ Radiotherapy is a non-invasive treatment with known side effects, the most frequently reported cataract (12.1%) and dry eye (8.5%)^25^, that are manageable through time. For example, cataract can be treated with surgery and the more common dry eye disease with artificial tears. The suggested radiotherapy doses range from 24 Gy up to 30 Gy.^17^ More than half of our patients were treated with 30 Gy, since the conclusions of a phase III trial of safely lowering the dosage to 24 Gy for indolent lymphoma was released in 201132 and our observation and treatment period of included patients began long before that.

Local control rate of 96.6% in our study is in line with Yen’s analyses (95.9% for studies of MALT lymphomas and 93.1% for studies that included data on multiple subtypes of lymphoma).^25^ Of note, our study included both multiple histologic subtypes of lymphoma and all OAL locations. Five-year overall survival rate in our study was 80.1% similar to Yen’s pooled analyses (78.9%).^25^ Half of our patients died due to causes unrelated to lymphoma, they were also significantly older than the rest of the study group, which underlines the importance of choosing the proper treatment or even active surveillance strategy with regard to patient’s preferences and comorbidities. The higher lymphoma-specific survival compared to the overall survival in our study shows that these patients are elderly, having other comorbidities that are more commonly the cause of death than lymphoma itself. Furthermore, we observed also a relatively high proportion of spontaneous remissions locally (54.5%, 6/11) in the active surveillance strategy group, suggesting that not all patients necessarily need the treatment immediately at presentation.

There is also an option of treating with only 4 Gy in 2 fractions for localized disease as reported by de Castro *et al*.^33^ Park *et al*.^34^ started a prospective phase II trial of 4 Gy in 2 fractions in stage I patients. Complete remission was observed in 11 lesions and partial remission in 6 of the 17 included lesions, respectively. The lesions with partial response were further treated to 24 Gy. Therefore, the future perspective for localized lesions is to reduce and adapt the radiation treatment if possible while still preserving excellent remissions and reducing the side effects.^34^

In Tanimoto’s study with a similar follow up time as in our study^35^, 69% of patients with MALT lymphoma did not need any treatment for localized disease. Similar to Tanimoto’s study, our active surveillance strategy group was comprised of predominately MALT histology. Even though the active surveillance group was not older than the treated group, we feel obliged to stress the importance of a careful evaluation of treatment strategy, comorbidities and patient’s preferences to avoid unnecessary additional complications caused by treatment.

Aggressive lymphomas (DLBCL, MCL, peripheral T-cell lymphoma, not otherwise specified) were treated according to guidelines with systemic treatment resulting in outcomes that are in line with reported histological subtypes.^36,37^ Considering the fact that low grade lymphomas are usually incurable and the recurrence of the disease is expected, we need to underline that there were only two cases of recurrence in periocular area, that have been already treated previously. Our 5-year overall survival rate and lymphoma-specific survival rate are high (80.1%; 87.2%), but again, as reported, OALs are known for high survival rates.^16,24,38^

Patients with localized disease (eye only) were in our study analyzed separately. In this group of patients, a higher 5-year overall (91.1% compared to 80.1%) and lymphoma-specific survival rate (100% compared to 87.2%), as well as a higher number of complete remissions (70.3% compared to 56.9%) and a lower number of systemic recurrences (24.3% compared to 43.1%) were registered. It can be therefore concluded that patients with a localized disease have a better outcome compared to patients with secondary OAL.

Ocular adnexal lymphomas comprise a group of heterogeneous diseases. Their outcomes vary in relation to patient’s age and lymphoma type. Individual treatment decisions are mandatory to tailor the treatment suitable for each patient. Radiotherapy has proven to be a highly efficient treatment with an excellent local control rate while systemic treatment should be reserved for disseminated disease.
